# Necrotizing Pancreatitis Infected with *Stenotrophomonas maltophilia*: An Emerging Rare Multidrug-Resistant Organism

**DOI:** 10.1155/2023/8071158

**Published:** 2023-06-23

**Authors:** Sophia Dar, Nooraldin Merza, Maryam Haider, Yousaf Zafar, Noren Din, Rosario Ligresti, Rani Sebti

**Affiliations:** ^1^Department of Internal Medicine, Hackensack University Medical Center, Hackensack, NJ, USA; ^2^Department of Medicine, Wayne State University, Detroit, MI, USA; ^3^Department of Internal Medicine DMC, Wayne State University, Sinai Grace Hospital, Detroit, MI, USA; ^4^Department of Medicine, University of Mississippi Medical Center, Oxford, MS, USA; ^5^Division of Gastroetnerology, The Pancreas Center, Hackensack University Medical Center, Hackensack University School of Medicine, National Pancreas Foundation, Hackensack, NJ, USA; ^6^Department of Medicine, Hackensack University School of Medicine, Division of Infectious Disease, Hackensack University Medical Center, Hackensack, NJ, USA

## Abstract

*Stenotrophomonas maltophilia *(SM) is a multidrug-resistant, Gram-negative (GN) bacillus that is an increasingly recognized nosocomial and environment pathogen. It is intrinsically resistant to carbapenems, a drug commonly utilized in the management of necrotizing pancreatitis (NP). We report a 21-year-old immunocompetent female with NP complicated by pancreatic fluid collection (PFC) infected with SM. One-third of patients with NP will develop infections by GN bacteria, while broad-spectrum antibiotics, including carbapenems, cover most infections, trimethoprim-sulfamethoxazole (TMP-SMX) is the first-line treatment antibiotic for SM. This case is critical because it highlights a rare pathogen that should be considered a causal pathogen in patients who do not respond to their care plan.

## 1. Introduction

SM is an aerobic, multidrug-resistant, GN, and ubiquitous bacillus. It is an environmental pathogen that has become an increasingly important cause of nosocomial infections [1–4]. Its pathogenicity has led to an escalating number of cases in immunocompromised patients, patients on mechanical ventilation, and patients subject to invasive procedures [1–4]. SM can lead to several medical conditions including soft tissue infections, endocarditis, pneumonia, meningitis, and peritonitis, but not limited to bacteremia [1–4]. SM is consistently resistant to multiple antibiotics, including carbapenems, the latter being a drug commonly used in the management of NP [3]. Traditionally, the drug of choice (DOC) of SM infections has been with the bacteriostatic compound TMP-SMX [3].

We report a patient with NP complicated by a PFC infected with SM. The patient was successfully treated with endoscopic interventions and antibiogram-based antibiotic therapy. To our knowledge, there are only a few documented cases of PFC infected by SM, but none in an otherwise immunocompetent patient whose likely source was the community. Physicians treating patients with severe NP, especially in those with extended hospital stay in the intensive care unit (ICU), multiple comorbidities, exposure to prosthetic devices or other equipment, and prolonged exposure to broad-spectrum antibiotics such as carbapenems should be aware of SM as a potential pathogen in their patients.

## 2. Case Presentation

A 21-year-old female presented to the emergency department (ED) with one day of vomiting and progressive and severe abdominal pain (AP) localized to the right upper quadrant and epigastric pain radiating to the back. She had nausea for the prior few weeks, which she had attributed to her pregnancy. She also had darker-colored urine and clay-colored stools. Her past medical history was significant for a spontaneous vaginal delivery two weeks prior and primary hypothyroidism appropriately managed with medical therapy (TSH-3.210 *μ*IU/mL/T4-0.97 *μ*g/dL on 100 mcg Synthroid). On physical examination, the patient is obese (BMI 32.12 kg/m^2^), temperature of 98.3 F, heart rate of 63 beats per minute, and blood pressure of 128/66 mm·Hg. She appeared mildly jaundiced without any stigmata of chronic liver disease. Abdominal examination revealed a soft and nondistended abdomen positive for epigastric tenderness. The patient denied any history of alcohol abuse. Admission laboratory values were hemoglobin of 14.2 gm/dL, leucocyte count 15.6/mcl, platelet count 371/mcl, serum glucose 139 mg/dL, serum aspartate transaminase (AST) 331 IU/L, serum alanine transaminase (ALT) 522 IU/L, total bilirubin 6.2 mg/dL, blood sugar ranging 93–117 mg/dL, triglycerides 80 mmol/L, amylase 1847 U/L, and lipase >8000 U/L.

**Figure 1:**
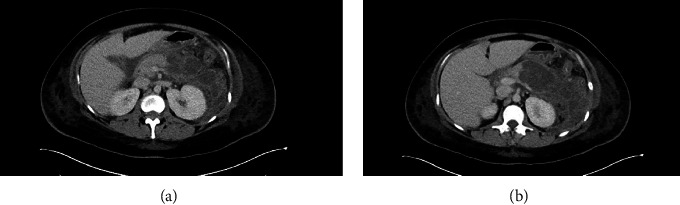
(a, b) CT scan showing the pancreatic fluid collection measuring 13.2 × 5.5 cm in cross-section and 12.8 cm in craniocaudal length.

The patient subsequently had magnetic resonance imaging (MRI) with magnetic resonance cholangiopancreatography (MRCP) revealing severe NP involving the body and predominantly the tail of the pancreas, cholelithiasis without biliary ductal dilation or visible stones, and ascites with small bilateral pleural effusions. The patient was initially treated with intravenous fluids (IV), IV piperacillin-tazobactam (PT), and kept NPO (nothing by mouth). The patient became febrile a few days into admission leading to a computerized tomography (CT) scan done on day (D) 4, which showed extensive NP and peripancreatic fluid in the retro gastric region. Despite antipyretic medications, she continued to be febrile. On D8, a repeat CT scan showed NP with a large developing PFC collection measuring 13.2 × 5.5 cm in cross-section and 12.8 cm in craniocaudal length (Figure 1). The patient was then started on a seven-day IV meropenem and vancomycin course. A CT-guided fine-needle aspiration done on D10 withdrew five cc of serosanguinous fluid, which was found to be sterile. The patient's hospital course continued to be complicated by fevers and AP. On D16, the PFC had reached a 13 cm anteroposterior diameter, replacing the pancreatic body and tail seen. On D17, the patient started to feel better and demanded to go home. She was informed that the pseudocyst was quite large and would not resolve without intervention.

On day 27, she returned as her symptoms returned and had an esophago-gastroduodenoscopy performed first, before stent placement, using a gastroscope (GIF–H190; Olympus, Tokyo, Japan). The esophagus and stomach were cleared of any retained particulate matter with water lavage and suction. The routine forward-view examination allowed the endoscopist to evaluate for other possible pathologies and identify any areas with extrinsic compression.

Then, a linear echoendoscope (GF–UCT180; Olympus, Tokyo, Japan) was used for the echo-endoscopic portion of the procedure. Under sonographic guidance, the PFC was carefully evaluated for an internal vessel, solid debris, and location in relation to the gastric wall. An effacement between the gastric and cystic walls with <1 cm distance and absence of vascular structures in the needle trajectory was confirmed under sonographic guidance before stent placement.

An ELAMS (Boston Scientific, USA) was placed by puncturing into the fluid collection either directly under a “pure-cut” generator setting or over the guidewire after a fine-needle aspiration needle pierced the cyst. The inner flange was deployed first under endosonographic guidance. The opened flange was then pulled into the cyst wall, pressing against its opposing stomach wall. Then, the outer flange was deployed inside the gastric lumen, confirmed by both endoscopy and fluoroscopy. Carbon dioxide was used for insufflation (UCR, Olympus, Japan) during the entire length of the procedure.

**Figure 2:**
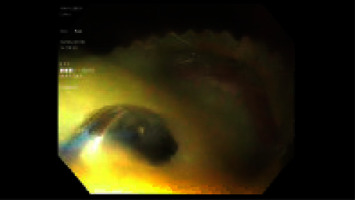
Endoscopic image showing pus drainage after dislodging necrotic debris noted to occlude the cystogastrostomy tract.

Puncturing the gastric wall, stent deployment, and final stent position was guided and confirmed under EUS with additional fluoroscopic guidance. Fluoroscopy was used primarily to monitor the stent deployment process and evaluate the stent position, while other utilities such as assessing the needle trajectory while puncturing the fluid collection, aiding in guidewire placement before stent placement, and documenting the stent position for subsequent evaluation when stent migration was suspected were also applied. Cystic fluid was aggressively suctioned to minimize aspiration risk. 600 mL of necrotic debris was noted to drain (Figure 2). The patient was discharged home.

On D31, the patient returned to the ED complaining of fever (101.1 F), AP radiating to her back, and vomiting. A CT scan was performed and showed an interval decrease in size of the sizeable peripancreatic pseudocyst, now measuring 8.7 mm maximally anteroposterior diameter and interval air related to communication with the tip of the stomach. She was then readmitted for an endoscopic pancreatic necrosectomy (PN) and started on ceftriaxone 2 g ever 12 hours. On D35, extensive PN was performed, and the walled-off necrotic cavity was irrigated with 1.5% hydrogen peroxide. The cystogastrostomy was stented concentrically with the AXIOS stent (10°F). Antibiotics were also switched to IV meropenem and IV vancomycin empirically for another seven-day course.

Gram stain of the PFC revealed the presence of Gram-positive cocci in pairs and chains and GN bacilli. The fluid cultures grew methicillin-resistant*Staphylococcus aureus* (MRSA) and 2.5 × 10^7^ cfu/mL SM. The antibiotic susceptibility report (antibiogram) revealed that MRSA was sensitive to clindamycin, TMP-SMX, and vancomycin. SM was susceptible to TMP-SMX. Antibiotic therapy was then narrowed to TMP-SMX (800 − 160 mg 4 times a day) and vancomycin (1 g every 12 hours) on D37. The patient improved on these antibiotics and was no longer febrile and no longer had significant AP. On D48, the patient was discharged on Linezolid 600 mg every 12 hours for a complete antibiotic course of 21 days.

She underwent three more endoscopic necrosectomies with hydrogen peroxide irrigation. The AXIOS stent was removed on the patient's third endoscopic necrosectomy at D60. One month after the last endoscopic pancreatic drainage, the patient underwent laparoscopic cholecystectomy for acute gallstone pancreatitis. Imaging at this time revealed the gallbladder to be thickened and contracted with cholelithiasis and resolution of the pancreatic pseudocyst.

## 3. Discussion

SM is a more commonly known as a nosocomial pathogen but is also a rising cause of community-acquired infections. SM is commonly associated with respiratory tract infections, bacteremia, biliary sepsis, endocarditis, meningitis, and urinary tract infections but is also associated with other multisystem infections [1–4]. The literature review showed cases infected with SM as one of the polymicrobial infections of the respiratory tract of cystic fibrosis [5]. Cases of community-acquired SM infections have been reported, but the incidence of hospital-acquired SM infections has been increasing, particularly in the immunocompromised population [2–4]. In both nosocomial and environmental settings, the organism has been known to be isolated from aqueous associated sources [3]. These include sources such as animals, lakes, hemodialysis water, and even dialysate samples [3]. One key virulence factor is its ability to use its polar flagella to form a biofilm [3]. The biofilm allows it to adhere to plastics and find itself on the surfaces of intravenous (IV) cannulae, prosthetic devices, and even nebulizers [3]. There are reports of SM infecting the biliary tree, but it is an unusual cause of necrotizing pancreatitis with infected pancreatic fluid collections [6]. However, one study compared nonzymogenic with zymogenic bacteria as a cause of pancreatitis and pancreatic necrosis and revealed an increased infection rate of nonzymogenic bacteria (*Pseudomonas aeruginosa*, *Acinetobacter baumannii*, and *Stenotrophomonas maltophilia*) compared to zymogenic bacteria (*Klebsiella pneumoniae*, *Escherichia coli*, and *Enterobacter cloacae*) (*P* < 0.01) [7]. The molecular and cellular mechanisms essential for its survival, persistence, and pathogenesis are likely the reason for its ability to persist in nutrient-poor regions such as fluid collections [3]. There are some reports in the literature of similar pathogens that have been pathogenic likely by their ability to form a biofilm; however, in this case, we do not believe the patient encountered any surgical instrumentation exposing her to any such pathogens.

The source of the patient's infection remains unclear. She could have acquired the pathogen from her environment, and it is still possible that it somehow colonized her biliary tree. However, she likely developed the infection [11] due to selective pressure from multiple antibiotic courses, which led to SM being the predominant infection. We believe that it was unlikely to be colonization as she had not had any surgical procedures that would have predisposed her to colonization prior to the diagnosis. She also had a significant amount of the bacteria in the pancreatic fluid and responded extremely well after the antibiotic treatment was changed to target the SM. During her hospitalization, she received one entire seven-day course of meropenem and vancomycin and numerous short courses of ceftriaxone and piperacillin-tazobactam. Had a more common pathogen been the cause for her pancreatitis, her disease course should have resolved after receiving multiple lines of first-line antibiotics. We believe the multiple courses of the antibiotics actually worsened the disease through selective pressure.

The patient noted that her symptoms of nausea started at the end of her pregnancy, although she attributed the symptoms to the pregnancy itself. She had a healthy delivery and was immunocompetent for the remainder of her illness. She had no prior history of serious medical diseases and had no family history of immune-compromising medical conditions. Her blood cell lines also remained within normal limits for the duration of her treatment. A few reported cases of biliary sepsis have been seen in the past postinstrumentation with biliary drainage catheters [6]. This may show that the organism has the potential to colonize the biliary tree. This patient, however, had no prior instrumentation that may have led to colonization. Her PFC may have become infected with SM and in her instance, become a true pathogen; conceivably, this patient may have developed an invasive infection due to SM as a result of selective pressure caused by the multiple courses of antibiotics that she received. Indeed, during her hospital stay, she received various courses of antibiotics, including two days of ceftriaxone, four days of PT, nine days of meropenem, and nine days of vancomycin before her culture returned positive for SM. Her antibiotics were narrowed to TMP-SMX and vancomycin.

This case is critical because it is the only well-documented case of documented SM infection of a PFC in an immunocompetent patient whose likely source was the environment. Through selective pressure from multiple antibiotics, it became a true pathogen. We expect that there have been numerous cases prior that have passed with this organism unidentified. It is essential to consider SM as a possible cause of infection of PFC in patients that initial broad-spectrum antibiotics do not show improvement. A human study investigating the levels of penetration of different broad-spectrum antibiotics found that ciprofloxacin, ofloxacin, imipenem, and pefloxacin provided sufficient tissue concentrations to reduce mortality and rates of infection [8]. These antibiotics cover the most common microbes identified in infected PN, which are *Escherichia coli* (20.0%) and *Enterococcus faecalis* and *faecium* (22.5% and 20.0%) [9]. Many cases, however, are shown to be polymicrobial [9]. SM is known for its resistance to multiple broad-spectrum antibiotics, including imipenem that current guidelines recommend using as the first line. As previously mentioned, SM is more sensitive to TMP-SMX alone or in synergistic combinations. Few previous studies showed that although trimethoprim-sulfamethoxazole is the drug of choice when studied *in vitro*, few other medicines, including ticarcillin-clavulanic acid, minocycline, some of the new fluoroquinolones, and tigecycline, are useful as well [10]. Considering there are only a few documented case reports, it is unnecessary to cover empirically for SM initially in these patients, but suspected infected PFC should be cultured and regularly reviewed in patients with progressively worsening clinical pictures. These patients may not have appropriate antibiotic coverage despite the broad-spectrum antibiotics, as in the case of our patient. A physician should have a low index of suspicion in patients whose clinical condition is deteriorating and whose patients have had a prolonged course of carbapenems.

## Data Availability

The data used to support the findings of this publication are included in this article and references.

## Additional Points

*Key Highlights*. (i) This patient is an unusual case of *Stenotrophomonas maltophilia* and emphasizes the need to explore rare infectious agents when the patient is not responsive to treatment. (ii) There is also an emphasis on treating with the most effective antibiotic for the infectious agent, which in this was trimethoprim-sulfamethoxazole. (iii) Interventional methods (such as, in this case, endoscopic necrosectomy) are essential for both source control and acquiring samples for culture and sensitivity.
